# Oxyresveratrol Inhibits R848-Induced Pro-Inflammatory Mediators Release by Human Dendritic Cells Even When Embedded in PLGA Nanoparticles

**DOI:** 10.3390/molecules26082106

**Published:** 2021-04-07

**Authors:** Salvatore Calogero Gaglio, Marta Donini, Piyachat Evelyn Denbaes, Stefano Dusi, Massimiliano Perduca

**Affiliations:** 1 Department of Biotechnology, University of Verona, Strada Le Grazie 15, 37134 Verona, Italy; salvatorecalogero.gaglio@univr.it (S.C.G.); pyachat@live.com (P.E.D.); 2 Department of Medicine, Section of General Pathology, University of Verona, Strada Le Grazie 8, 37134 Verona, Italy; marta.donini@univr.it

**Keywords:** dendritic cells, oxyresveratrol, cytokines, PLGA nanoparticles, inflammation

## Abstract

Oxyresveratrol, a stilbene extracted from the plant *Artocarpus lakoocha* Roxb., has been reported to provide a considerable anti-inflammatory activity. Since the mechanisms of this therapeutic action have been poorly clarified, we investigated whether oxyresveratrol affects the release of the pro-inflammatory cytokines IL-12, IL-6, and TNF-α by human dendritic cells (DCs). We found that oxyresveratrol did not elicit per se the release of these cytokines, but inhibited their secretion induced upon DC stimulation with R848 (Resiquimod), a well-known immune cell activator engaging receptors recognizing RNA viruses. We then investigated whether the inclusion of oxyresveratrol into nanoparticles promoting its ingestion by DCs could favor its effects on cytokine release. For this purpose we synthesized and characterized poly(lactic-co-glycolic acid) (PLGA) nanoparticles, and we assessed their effects on DCs. We found that bare PLGA nanoparticles did not affect cytokine secretion by resting DCs, but increased IL-12, IL-6, and TNF-α secretion by R848-stimulated DCs, an event known as “priming effect”. We then loaded PLGA nanoparticles with oxyresveratrol and we observed that oxyresveratrol-bearing particles did not stimulate the cytokine release by resting DCs and inhibited the PLGA-dependent enhancement of IL-12, IL-6, and TNF-α secretion by R848-stimulated DCs. The results herein reported indicate that oxyresveratrol suppresses the cytokine production by activated DCs, thus representing a good anti-inflammatory and immune-suppressive agent. Moreover, its inclusion into PLGA nanoparticles mitigates the pro-inflammatory effects due to cooperation between nanoparticles and R848 in cytokine release. Therefore, oxyresveratrol can be able to contrast the synergistic effects of nanoparticles with microorganisms that could be present in the patient tissues, therefore overcoming a condition unfavorable to the use of some nanoparticles in biological systems.

## 1. Introduction

Dendritic cells (DCs) are a heterogeneous cell population endowed with the ability to phagocytose antigens present in the extracellular environment. Subsequently, DCs undergo a process of maturation which enables them to present antigens to lymphocytes, thus activating the specific immune response [1,2,3]. Mature DCs produce chemical mediators that modulate the adaptive immune reaction and the inflammatory process to fight cancer cells and pathogen microorganisms. Among the most important mediators secreted by DCs there is IL-12, a fundamental cytokine activating natural killer cells and T lymphocytes [4,5], as well as IL-6 and TNF-α that stimulate the immune cells and are involved in the induction of the systemic acute phase reaction characterized by fever, headache, changes in the sleep-wake cycle, anorexia, nausea and emesis [6,7]. Therefore DCs play a fundamental role in regulating both inflammatory and adaptive immune response, and a decreased tolerogenic activity of these cells can promote pathological events such as hypersensitivity and autoimmunity [8,9]. Interestingly, DC treated with the polyphenol resveratrol during their differentiation have been reported to became tolerogenic cells showing decreased ability to stimulate T cells because of a reduced expression of co-stimulatory molecules and IL-12, and an increased secretion of the immune suppressive cytokine IL-10 [10]. Moreover oxyresveratrol, a polyphenol derived from *Artocarpus lakoocha* Roxb.heartwood, a plant known in Thai as ‘Ma-Haad’ and used in traditional medicine [11], has been shown to exert anti-inflammatory effects by downregulating the pro-inflammatory cytokine production in several experimental conditions [12,13,14,15]. However, the mechanisms by which polyphenols exert their anti-inflammatory activity remain to be fully elucidated and, as far as we know, no investigations have been performed on the effect of oxyresveratrol on the functional activity of human DCs. This prompted us to examine whether a mechanism by which oxyresveratrol blunts the inflammation would reside in the inhibition of human DC ability to produce pro-inflammatory cytokines.

Biodegradable polymeric nanoparticles have been extensively used as delivery systems of active components such as anticancer drugs, vitamins, proteins, peptides, and others. There are many advantages using such technology, since polymeric nanoparticles protect active compounds from degradation, improve their solubility, and promote controlled drug release and targeting [16]. However, the administration of nanostructures to patients is sometimes hindered by possible side-effects due to their encounter with the cells of the immune system, which protect the body from foreign intrusions. In fact, the interaction of some nanoparticles with immune cells, including DCs, can cause their activation and consequently adverse effects such as inflammation or allergy [17,18]. Many different polymers have been used to produce nanoparticles, but in particular poly(lactic-co-glycolic acid) (PLGA) has received broad interest because it is biocompatible, biodegradable and approved for human therapies both by the Food and Drug Administration (FDA) and the European Medicines Agency (EMA), [19,20,21]. Therefore, we investigated whether the insertion of oxyresveratrol into PLGA nanoparticles would modulate the effects of this polyphenol on DCs.

## 2. Results

### 2.1. Synthesis and Characterization of PLGA Nanoparticles

The aim of our investigations was to assess whether oxyresveratrol alone or encapsulated into polymeric nanoparticles would affect the release of proinflammatory cytokines by human DCs. First, we synthesized PLGA nanoparticles, and we loaded them with oxyresveratrol. We decided to focus our attention on the encapsulation in PLGA via single emulsion evaporation method. As illustrated in Table 1, we were able to obtain PLGA nanoparticles embedding oxyresveratrol showing a diameter quite similar to the one of empty PLGA nanoparticles, with an average size of 169.6 ± 3.5 and a polydispersity index (PDI) of 0.06 ± 0.02.

To further investigate the size distribution of these nanoparticles, Nanotracking and atomic force microscopy (AFM) analysis were also performed. Figure S1 shows that the nanoparticle shape was quite spherical and, as it is evident from Table S1, data obtained by using three different methods were in agreement and spanned in the same order of magnitude, even if the techniques were based on different physico-chemical properties.

In addition, the monodispersed trend was observed for both dynamic light scattering (DLS) and Nanosigtht diagram (Figure S2). The encapsulation of oxyresveratrol did not affect the size and size-distribution of the nanoparticles, but rather it made the ζ-potential less negative (−7.1 ± 0.5) than the one of empty nanoparticles (−9.6 ± 0.4) (Table 1); despite this slight difference, oxyresveratrol loaded nanoparticles showed a good colloidal stability as well.

**Figure 1:**
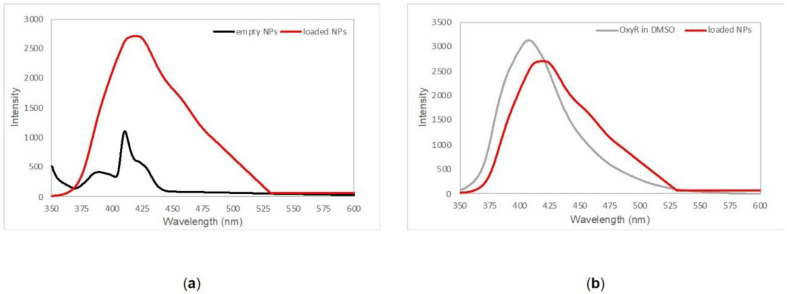
(**a**) Emission spectra of empty PLGA nanoparticles (black line) and oxyresveratrol-loaded PLGA nanoparticles (red line) collected in phosphate buffer saline pH 7.4. (**b**) Overlapping between free oxyresveratrol emission pattern in DMSO (gray line) and oxyresveratrol encapsulated in PLGA nanoparticles (red line).

The presence of oxyresveratrol in the PLGA nanoformulation was assessed by fluorescence spectroscopy. Figure 1a shows the emission pattern of loaded and empty nanoparticles: upon excitation at 335 nm, oxyresveratrol loaded nanoparticles exhibited a fluorescence spectrum different than the one shown by empty nanoparticles. Figure 1b illustrates that the emission peak around 410–420 nm of oxyresveratrol encapsulated in PLGA nanoparticles overlapped the emission spectrum of the free polyphenol in DMSO, and both these data are indicative of effective entrapment of oxyresveratrol into PLGA nanoparticles. The observed red-shift trend was probably due to an interaction with the hydrophobic part of the polymer that could affect the emission pattern.

The encapsulation efficiency for these nanoparticles was found to be close to 45% ± SD; very similar values, within the experimental error, were obtained using a direct method, based on the quantification of the oxyresveratrol loaded on the nanoparticles, and with the indirect method, where the bound fraction was estimated as the difference between the total amount added in the reaction buffer and the unloaded fraction still remaining after the reaction and the two sequent washing steps. The two similar values can be found in Table S2.

**Figure 2:**
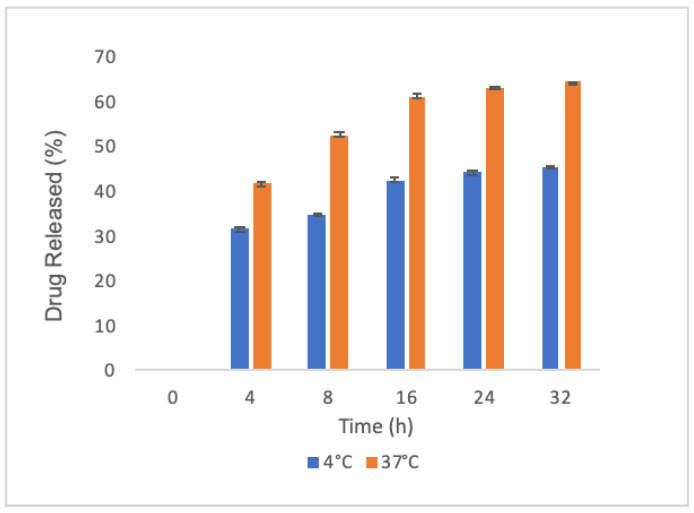
Oxyresveratrol release by PLGA nanoparticles: average cumulative data at 4 °C (blue bars) and 37 °C (orange bars) in phosphate buffer saline pH 7.4; each sample was collected at the indicated time points. Data were acquired in triplicate and are expressed as the mean value ± SD.

We then investigated whether PLGA nanoparticles released oxyresveratrol at various time points and temperatures (4 and 37 °C) by UV-Visible spectroscopy to understand the release profile over time in preserving conditions (4 °C) and at the temperature used during the interaction with DCs. We found that oxyresveratrol-loaded nanoparticles exhibited an initial burst release in the first four hours with a sustained trend in the next 28 h, reaching a value of 64% in 32 h (Figure 2). As expected, the trends at different temperatures were similar, even if the increase in temperature led to a curve with an enhanced slope.

### 2.2. Oxyresveratrol Inhibited the Release of Pro-Inflammatory Cytokines by R848-Stimulated DCs

**Figure 3:**
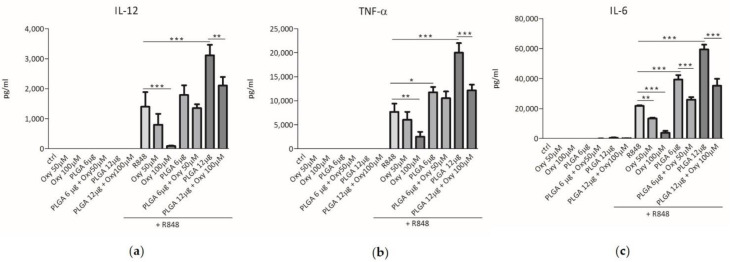
Effects of free or PLGA-conjugated oxyresveratrol on pro-inflammatory cytokine release by human dendritic cells (DCs). DCs were treated for 24 h with the indicated doses of free oxyresveratrol (Oxy), bare PLGA nanoparticles (PLGA) or oxyresveratrol-bearing PLGA nanoparticles (PLGA + Oxy). All the treatments were conducted in the absence or presence of 5 μM R848. The release of IL-12 (**a**), TNF-α (**b**) and IL-6 (**c**) in culture supernatants was evaluated by ELISA assay. The results are expressed as the mean value ± SD of four independent experiments. * *p* < 0.05, ** *p* < 0.01 *** *p* < 0.001 by two-way ANOVA followed by Bonferroni post-test.

The interaction between DCs and pathogenic microorganisms triggers the secretion of various cytokines that stimulate the inflammatory process and the immune response [22]. We then investigated whether free oxyresveratrol would be able to modulate the cytokine release elicited by DC challenge with R848, a molecule binding the toll like receptor (TLR)7 and TLR8, which are pattern recognition receptors (PRRs) sensing viral RNA and activating the immune cells [23,24]. For this purpose, blood monocyte-derived DCs were treated with 50 or 100 μM free oxyresveratrol, both in the absence or presence of 5 μM R848. After a 24 h incubation, the culture supernatants were collected and the cytokine secretion was analyzed by ELISA. Figure 3 shows that, at the above mentioned doses, oxyresveratrol did not trigger the release of IL-12, TNF-α, or IL-6 by DCs, but inhibited the R848-induced secretion of all these pro-inflammatory cytokines.

### 2.3. The Insertion of Oxyresveratrol in PLGA Nanoparticles Inhibited the Synergy between These Particles and R848 in Induction of Cytokine Release by DCs

We previously demonstrated that the PLGA nanoparticles produced in our laboratory were efficiently internalized by human monocyte-derived DC [25]. Therefore, we wondered whether incorporation of oxyresveratrol into these nanoparticles, that can promote its internalization by cells, would influence the effects of this polyphenol on cytokine secretion by DCs. First, we checked whether unloaded PLGA particles prepared in our laboratory would affect the cytokine release by human DCs. Figure 3 shows that the incubation of resting DCs with 6 or 12 μg of bare PLGA particles did not trigger cytokine release. However, unloaded PLGA particles significantly and dose-dependently increased the cytokine secretion by R848-stimulated DCs (Figure 3). These results demonstrate that the PLGA nanoparticles synthesized following our protocol are unable to interfere per se with human DC activation mechanisms, but can synergize with agonists of TLR7 and TLR8 in induction of pro-inflammatory cytokine secretion. The latter event reminds the so-called “priming effect”, that is the ability of some agents to induce a hyper-responsiveness of immune cells to other stimuli [26,27]. Figure 3 also shows that the challenge of unstimulated DCs with 50 and 100 μM oxyresveratrol enclosed into 6 and 12 μg of PLGA nanoparticles respectively, did not trigger cytokine secretion. Interestingly, oxyresveratrol encapsulated into PLGA nanoparticles significantly inhibited the synergistic effect of PLGA and R848 in the induction of IL-12, TNF-α and IL-6 release by DC (Figure 3). Therefore, oxyresveratrol insertion into nanoparticles can reduce an unwanted pro-inflammatory “priming effect” of PLGA particles on R848-induced cytokine release by DCs.

### 2.4. Evaluation of Oxyresveratrol and PLGA Nanoparticle Toxicity on Human DCs

**Figure 4:**
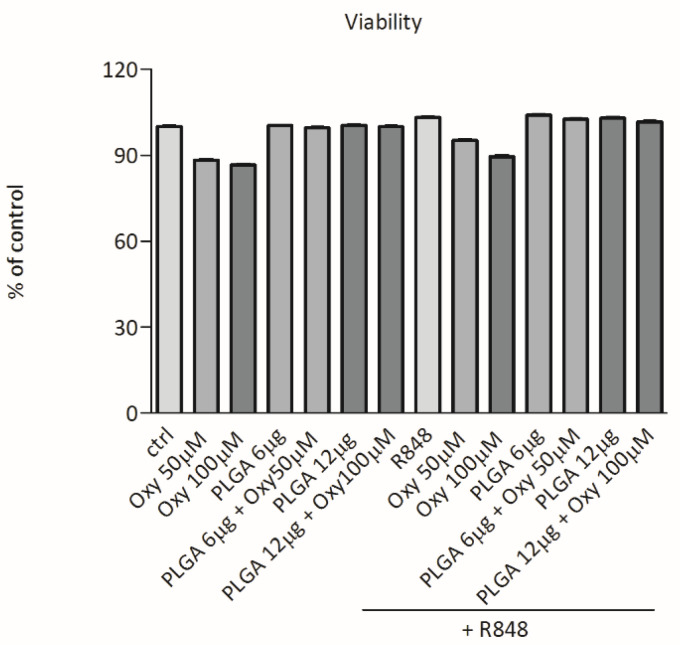
Effects of free oxyresveratrol and oxyresveratrol-bearing PLGA nanoparticles on DC viability. DCs were treated or not treated (ctrl) with the indicated doses of free oxyresveratrol (Oxy), unloaded PLGA nanoparticles (PLGA), or oxyresveratrol-bearing PLGA particles (PLGA + Oxy) for 24 h, followed by 1 h incubation with WST. Values are expressed as the percentage of WST reduction relative to untreated cells (designated as 100%). Data are means ± SD of four experiments.

To exclude that our results on cytokine release could be due to toxic effects we examined whether oxyresveratrol alone or inserted into PLGA nanoparticles would affect the DC viability. For this purpose, DCs were challenged with free oxyresveratrol, bare PLGA nanoparticles, or oxyresveratrol-loaded PLGA nanoparticles in absence or presence of R848 as in the experiments depicted in Figure 3 and cell viability was assessed using the Cell Proliferation Reagent WST-1 assay. Figure 4 shows that in these experimental conditions the DC viability was not or only slightly altered.

## 3. Discussion

Many attempts have been made to identify new molecules able to blunt the inflammatory response in absence of the harmful side-effects frequently observed following administration of the currently used glucocorticoids and non-steroid anti-inflammatory drugs [28,29]. Oxyresveratrol is a polyphenol derived from a plant, *Artocarpus lakoocha* Roxb. (Moraceae), which has been used in Thai traditional medicine as an antioxidant and to treat inflammatory and parasitic diseases [30,31,32]. Recently, many studies have been performed to investigate the pharmacological effects of oxyresveratrol: in particular, it has been reported to suppress lipopolysaccharide-induced inflammatory response in murine models [31,33,34] and to exert its anti-inflammatory effects mainly by downregulating the release of various cytokines [12,13,14,15]. As far as we know, no investigations have been made on the effect of oxyresveratrol on the functional activity of human DCs, which play an essential role in activation of inflammation and immune reaction [1,2,3]. In this paper we report the results of investigations aimed at clarifying whether a mechanism by which oxyresveratrol inhibits the inflammation resides in a decreased secretion of pro-inflammatory cytokines by DCs. Here we show that oxyresveratrol suppressed the secretion of IL-12, TNF-α and IL-6 by human DCs stimulated with R848, an agonist of TLR 7 and TLR 8 which recognize single stranded RNA viruses such as Influenza, Sendai, Coxackie B, HIV and HCV [23,24]. Therefore, cell stimulation with R848 mimics the natural interaction between DCs and some pathogen viruses, and the results obtained suggest that oxyresveratrol could be a good tool to mitigate the inflammatory response elicited by these microorganisms. It is worth to emphasize that here we show that oxyresveratrol inhibited the R848-induced secretion of cytokines which play a fundamental role in activation of the inflammatory process. In fact, IL-12 stimulates T cells and natural killer cells to produce IFN-γ, which in turn widens the immune and inflammatory response [4,5,35], TNF-α mediates basal inflammatory events such as edema, leukocyte adhesion to epithelium, oxidative stress, and fever [6], and IL-6 activates, among others, acute phase responses, B lymphocyte maturation, and T cell functions [7]. Therefore, oxyresveratrol might be a useful tool to treat autoimmune and chronic inflammatory diseases, such as inflammatory bowel diseases, psoriasis, ankylosing spondylitis, multiple sclerosis, asthma, Crohn’s disease, ulcerative colitis, Alzheimer’s diseases and rheumatoid arthritis, in which IL-12, TNF-α, and IL-6 have been found to play an important role [7,36,37,38,39].

Then we wondered whether the effects of oxyresveratrol on R848-stimulated DCs could be enhanced by its insertion into particles that promote its internalization by DCs. It is well known that PLGA particles are efficiently ingested by human DCs [40,41,42]. Therefore, we synthesized empty and oxyresveratrol-loaded PLGA nanoparticles showing similar dimensions, both resulting in a monodispersed suspension in phosphate buffer saline (PBS), and having good colloidal stability. The encapsulation efficiency was close to 45%, and the release of oxyresveratrol by nanoparticles at 37 °C reached 64% in 32 h, ensuring to oxyresveratrol a good biodisponibility in the 24 h of exposure to dendritic cells. We previously showed that the PLGA nanoparticles produced in our laboratory were easily internalized by human monocyte-derived DCs, as demonstrated by confocal microscopy experiments [25]. Therefore, these PLGA nanoparticles represented a very good tool to carry oxyresveratrol inside the DCs.

The use of particles for medical purposes is often hampered by the ability of some of them to activate immune cells, thus leading to pro-inflammatory effects and adverse reactions such as fever, allergy or autoimmunity [17,18,43]. Therefore, we assessed whether PLGA nanoparticles produced in our laboratory induced per se DC activation. Here we show that both bare and oxyresveratrol-loaded PLGA nanoparticles were unable to activate cytokine release by resting DCs suggesting that these particles are biologically inert. However, bare PLGA enhanced the cytokine release triggered by DC stimulation with R848. We do not know the reasons of this unexpected pro-inflammatory effect. It might be due to the ability of PLGA nanoparticles to interact with unknown cellular targets able to cooperate with TLR7- and TLR8-dependent pro-inflammatory signals. As an alternative, the activation of phagocytosis mechanisms to engulf the PLGA particles might elicit pathways which collaborate with those induced by R848 to enhance cytokine release. This synergistic effect reminds the event named “cell priming”, characterized by the ability of some agents to induce an hyper-responsiveness of leukocytes to other stimuli if simultaneously or consequently added [26,27]. In this regard, it has been previously demonstrated that the microbial chemoattractant formyl-methionine-leucil phenylalanine (f-MLP) synergized the ability of ORMOSIL nanoparticles to induce the release of cytokines by human leukocytes [44]. Moreover, it has been reported that simultaneous addition of LPS and porous silicon-TiO_2_ microparticles was much more effective than stimulation with LPS alone to induce IL-12 and TNF-α secretion by human DCs [45]. Therefore, a synergistic effect between nanoparticles and molecules of microorganisms seems not to be a rare and unexpected result, although it is scantly investigated by researchers dealing with nanostructures. Whichever is the case, here we show that association of oxyresveratrol to PLGA nanoparticles blunted the PLGA nanoparticle-dependent enhancement of cytokine release by R848 treated DCs, indicating that this polyphenol maintains its anti-inflammatory properties also once conjugated to a nanostructure and, interestingly, that it could render a nanostructure less dangerous, in particular when administered in the presence of some microorganisms such as viruses. The mechanism by which oxyresveratrol inhibits the secretion of cytokines by human DCs remains to be clarified. However, on the basis of previous reports showing that this polyphenol decreased the release of cytokines from HMC3 human microglial and RAW 264.7 murine macrophage cell lines through inhibition of phosphatidylinositol 3-kinase [15,31], it is conceivable that a similar mechanism might also take place in human DCs.

In summary, our data indicate that a nanoparticle such as PLGA, having no apparent intrinsic pro-inflammatory activity, could participate to activation of pro-inflammatory events when administered in the presence of other unexpected agents, such as pathogens or their derivatives. At present, we have no explanation of the reason of this strange behavior, but the obtained results indicate that the interactions taking place among immune cells, nanostructures and microorganisms can be more complex than expected. The identification of the mechanisms involved in this effect went beyond the scope of the present work and will be the matter of future studies. Nevertheless, our data highlight the importance of an accurate and complete testing of both loaded and unloaded nanostructures in combination with products of microorganisms to avoid unwanted side-effects once they are administered to patients. Importantly, here we show that oxyresveratrol is a very good anti-inflammatory agent, which inhibits pathogen-induced inflammatory events, but can also mitigate an eventual synergistic effect between nanostructures and products of microorganisms when it is enclosed into such nanostructures. We have previously shown that PLGA-associated α-bisabolol, a natural sesquiterpene found in the oil of *Matricaria chamomilla*, decreased the secretion of cytokines by lipopolysaccharide (LPS)-stimulated human DCs [25]. These results, combined with those reported here, indicate that PLGA nanoparticles represent a good tool for carrying plant-derived molecules in order to inhibit the pro-inflammatory activity of DCs.

## 4. Materials and Methods

### 4.1. Materials

RPMI 1640 and low-endotoxin FBS were obtained from Lonza (Walkersville, MD, USA). Recombinant human GM-CSF and human IL-4 were purchased from Miltenyi Biotec (Bergisch Gladbach, Germany); Flow cytometric analysis was performed using the following mouse anti-human antibodies: CD83 (HB15e) and CD1a (HI149) (Becton Dickinson, San Jose, CA, USA); CD80 (2D10), CD86 (T2.2), HLA-DR (L243) and CD14 (M5E2) (Biolegend, San Diego, CA, USA). PLGA (poly[DL-lactide-co-glycolide] 50:50 lactide-glycolide ratio, CAS 26780-50-7), PVA (poly[vinyl alcohol], CAS 9002-89-5), Acetone (≥99% purity, 1.00013), Dimethyl sulfoxide (DMSO, ≥99% purity D-5879), Oxyresveratrol (≥97% purity, 91211) were purchased from Sigma-Aldrich, (St. Louis, MO, USA).

### 4.2. Preparation of PLGA Nanoparticles

The protocol used for the production of PLGA nanoparticles loading oxyresveratrol is based on single emulsion-evaporation method, under sterile conditions at 20 °C [19,46]. 10 mg of the polymer and 5 mM (1.22 mg) of oxyresveratrol from *Artocarpus lakoocha* heartwood were co-dissolved in 1 mL of organic solvent (95% Acetone and 5% DMSO); the obtained organic phase was added dropwise under stirring (2000 RPM) to 10 mL of 1% polyvinyl alcohol (PVA) aqueous solution and left overnight to evaporate the organic phase. Afterwards, the preparation was pelleted at 4 °C 11,000 rpm for 20 min (Eppendorf Centrifuge 5804R) and nanoparticles were collected and washed twice with 10 mL of Milli-Q water. Finally, purified nanoparticles were re-suspended in 1 mL of phosphate buffer saline solution pH 7.4 for the subsequent analysis and storage at 4 °C, otherwise freeze-dried. Empty PLGA nanoparticles were prepared with the same protocol avoiding the addiction of oxyresveratrol to the organic phase.

### 4.3. Size and ζ-Potential Characterization

Size and ζ-potential of PLGA nanoparticles were estimated at 25 °C using a Nano Zeta Sizer ZS (ZEN3600, Malvern Instruments, Malvern, Worcestershire, UK). Samples were resuspended in PBS, being used as a stock suspension, and were diluted 10 times from the stock solutions in PBS for size determination and into in 10 mM NaClO_4_ pH 7.5 for ζ-Potential measurements; data were collected in triplicate and analyzed by the ZetaSizer software (version 7.10).

To further support DLS data, a Nanosight tracking analysis was performed on PLGA(Oxy) NPs and empty PLGA nanoparticles (Malvern NanoSight NS300, Malvern Panalytical Ltd, Malvern, UK). Due to the high concentration, each sample was diluted 5000 times in PBS; 1498 frames divided in 3 runs of 60 s were recorded at camera level of 13 and the analysis were performed with a detection threshold in the range 5–7. Finally, the number of particles/mL was estimated as well.

Moreover, pictures of atomic force microscopy of both nanoparticle types were acquired: 20 μL of each sample (prepared as in the above section) were loaded on a bracket covered by inert mica surface. After 20 min for solvent evaporation, the analysis was performed using a NT-MDT Solver Pro atomic force microscope (Moscow, Russia) with NT-MDT NSG01 golden coated silicon tip in semi contact mode with different scanning frequencies (3–1 Hz) in order to produce optimized AFM images. The microscope was calibrated by a calibration grating (TGQ1 from NT-MDT) in order to reduce nonlinearity and hysteresis in the measurements. Finally, images were processed with the Scanning Probe Image Processor (SPIPTM) program (Image Metrology ApS, version 5.13, Lyngby, Denmark) and a statistical study was performed to compare results to DLS and Nanosight data.

### 4.4. Spectroscopic Studies, Encapsulation Efficiency and Release Evaluation

To assess the presence of oxyresveratrol inside our nanoparticles the emission pattern was recorded upon excitation at 335 nm, the absorption wavelength of the polyphenol, by using a Jasco Spectrofluorometer FP-8200 (Easton, MD, USA).

To quantify the amount of the entrapped oxyresveratrol (Encapsulation efficiency (EE)), a direct method was used. PLGA(Oxy) NPs were dissolved in DMSO and the obtained solution was analyzed using a calibration curve (Supplementary Materials Figure S3a). Encapsulation efficiency (EE) was estimated using the following Equation (1):

(1) $$\mathrm{EE}\;\left(\%\right)\;=\;\frac{{\mathrm{OxyR}}_{\mathrm{loaded}}}{{\mathrm{OxyR}}_{\mathrm{fed}}}\;\times \;100$$

To confirm this data a second method has been employed as well: waste supernatants from the nanoparticle preparation were collected and analyzed by comparing data to a second calibration curve obtained in Milli-Q water (Figure S3b). OxyR_loaded_ was indirectly estimated by the following Equations (2) and (3) and finally EE was calculated as described above.

(2) $${\mathrm{OxyR}}_{\mathrm{loaded}}\;=\;{\mathrm{OxyR}}_{\mathrm{fed}}\;-\;{\mathrm{OxyR}}_{\mathrm{lost}}$$

(3) $${\mathrm{OxyR}}_{\mathrm{lost}}\;=\;{\mathrm{OxyR}}_{\mathrm{Supernatant}1}\;+\;{\mathrm{OxyR}}_{\mathrm{wash}1}\;+\;{\mathrm{OxyR}}_{\mathrm{wash}2}$$

To assess the ability of the nanoparticles to retain oxyresveratrol over the time, a release study was performed in 1 mL of PBS pH 7.4 at 4 and 37 °C. Samples were collected at different time intervals and replaced with an equal volume of media to maintain the sink condition. The released OxyR was quantified using a calibration curve obtained by UV-Visible spectroscopy at 335 nm.

### 4.5. DC Preparation and Culture

After written informed consent and upon approval of the ethical committee (Prot. N. 5626, 2 February 2012; Prot. n. 57182, 16 October 2019), buffy coats from the venous blood of normal healthy volunteers were obtained from the Blood Transfusion Centre of the University of Verona. Peripheral blood mononuclear cells were isolated by Ficoll-Hypaque and Percoll (GE Healthcare Life Science) density gradients and used as a source for immunomagnetic isolation of CD14-positive cells (Miltenyi Biotec GmbH). The purity of CD14+ cells was always greater than 98%, as determined by flow cytometry. To generate immature DCs, monocytes were cultured at 37 °C, 5% CO_2_ at 1 × 10^6^/mL in 6-well tissue culture plates (Greiner, Nurtingen, Germany) in RPMI-1640 supplemented with 10% FBS (<0.5 EU/mL; Sigma-Aldrich, (St. Louis, MO, USA), 2 mM l-glutamine, 50 ng/mL GM-CSF and 20 ng/mL IL-4. After 5 days, non-adherent immature DCs were harvested and characterized by flow cytometry as CD1a^high^, CD80^−^, CD83^−^, CD86^low^ and HLA-DR^low^. To induce cytokine release, DCs were stimulated for 24 h with 5 μM R848 (Invivogen).

### 4.6. Quantification of Cytokine Production

ELISA development kits purchased from Mabtech (Nacka Strand, Sweden) were used to assay the protein levels of IL-12 (p70) (range 6–600 pg/mL), IL-6 (range 10–1000 pg/mL) and TNF-α (range 4–400 pg/mL) in the cell culture supernatant, according to the manufacturer’s instructions. Briefly, DCs resting or activated with 5 µM R848 were treated with oxyresveratrol alone or encapsulated in PLGA nanoparticles, or with corresponding amounts of bare PLGA particles for 24 h, and then the supernatants were collected. Several dilutions of each supernatant were incubated for 2 h at room temperature protected from the light in appropriate assay plates (EIA/RIA Plate, 96 Well Half Area, Flat Bottom, High Binding purchased from Corning (Corning, NY, USA) previously coated overnight at 4 °C with 50 μL/well Capture Antibody and then blocked by addition of 100 μL/well Assay Diluent for 1 h at RT. Supernatants were discarded and Detection Antibody (50 μL/well) was added. After 1 h incubation at RT, Avidin-HRP (50 μL/well, 30 min) and subsequently Substrate Solution (50 μL/well, 15 min), were added. The reaction was stopped with Stop Solution. Every step of the above procedure was followed by appropriate plate washes. The plates were read at 450 nm with Victor3 1420 Multilabel Counter PerkinElmer. A standard curve was prepared by serial dilution of standards and used for determining the cytokine concentrations in supernatants.

### 4.7. Cell Viability Evaluation

Cell viability was assessed using the Cell Proliferation Reagent WST-1 assay (Roche Diagnostics GmbH, Mannheim, Germany) according to the manufacturer’s instructions. DCs resting or activated with R848 were treated with oxyresveratrol alone or encapsulated in PLGA nanoparticles, or with corresponding amounts of bare PLGA particles for 24 h. After treatment, cell supernatant was removed and 50 µL of pre-warmed fresh complete medium were added to the cells and to 3 empty wells (Blank). A 2× WST solution was freshly prepared by dilution of the 10× WST reagent in the complete medium and a volume of 50 μL was dispensed in the wells and blank. The plate was incubated for 60 min. The absorbance (OD) of the samples was measured using a Victor3 multilabel reader (PerkinElmer, Shelton, CT, USA) at 450 nm.

### 4.8. Statistical Analysis

Data are expressed as means + SD. Statistical analyses, including two-way ANOVA followed by Bonferroni post-test, were performed with GraphPad Prism 5 (GraphPad Software, Inc., San Diego, CA, USA).

## 5. Conclusions

An important contribution of our findings is the concept that interaction of nanostructures with immune cells could lead to unexpected effects, in particular if microorganisms are presents in the tissues of patients to whom nanoparticles are administered. As evidenced when analyzing the IL-12, IL-6 and TNF-α release, PLGA nanoparticles can potentiate the pro-inflammatory effect exerted by R848, an agonist recognizing some virus-sensing receptors, on human DCs. However, the inclusion of oxyresveratrol into PLGA nanoparticles had an inhibitory effect on this synergistic mechanism, making the nanoparticles less dangerous. These data, although limited to IL-12, IL-6 and TNF-α, which are anyway fundamental mediators of inflammation [4,5,6,7], indicate that oxyresveratrol, in addition to carrying out an anti-inflammatory effect on virus-stimulated DCs, once inserted into particles can render them more biocompatible. In conclusion here we show that oxyresveratrol represents a useful molecule to inhibit the activity of human DCs. Owing to the essential role of DCs in the regulation of the inflammatory and immune reaction, we believe that the results of our investigation could be useful for a possible future translation into the clinical practice of anti-inflammatory and immunosuppressive therapy.

## Figures and Tables

**Table 1:** Dynamic light scattering and ζ-potential data of unloaded (EMPTY) and oxyresveratrol-loaded (PLGA(oxy)) poly(lactic-co-glycolic acid) (PLGA) nanoparticles. The results are expressed as the mean value ± SD of three independent measures on three replica samples.

Nanoformulation	Particles Size (nm)	Polydispersity Index	Ζ-Potential (mV)
EMPTY	170.2 ± 2.5	0.049 ± 0.040	−9.6 ± 0.4
PLGA(Oxy)	169.6 ± 3.5	0.06 ± 0.02	−7.1 ± 0.5

## Data Availability

No new data were created or analyzed in this study. Data sharing is not applicable to this article.

## Supplementary Materials

The following are available online, Figure S1: AFM image of a single oxyresveratrol-loaded PLGA nanoparticle with a resolution of 2.5 × 2.5 μm (a). Image of several single loaded nanoparticles and an aggregate with a resolution of 20 × 20 μm (b). The images were collected in intermittent mode. Figure S2: DLS Number diagram (a) and Nanosight tracking analysis diagram (b) of oxyresveratrol-loaded PLGA nanoparticles. Measurements where performed in triplicate. Figure S3: Calibration curve for oxyresveratrol in DMSO (a). Calibration curve for oxyresveratrol in aqueous solution (b). Table S1. Comparison between DLS, AFM and Nanosight tracking analysis data for empty and oxyresveratrol loaded nanoparticles. Table S2. Comparison between encapsulation efficiency value calculated directly breaking the nanoparticles and the same value indirectly estimated.

## Sample Availability

Samples of the compounds are available from the authors.
