# Supplementary material for: Oxyresveratrol Inhibits R848-Induced Pro-Inflammatory Mediators Release by Human Dendritic Cells Even When Embedded in PLGA Nanoparticles
Source: Molecules. 2021 Apr 7;26(8):2106. doi: 10.3390/molecules26082106 (PMC8067564; doi:10.3390/molecules26082106)
Supplement: Supplementary file 1 [file molecules-26-02106-s001.pdf]

Supplementary Information for

Oxyresveratrol inhibits R848-induced pro-inflammatory mediators release by human dendritic cells even when embedded in PLGA nanoparticles

Salvatore Calogero Gaglio [1], Marta Donini [2], Piyachat Evelyn Denbaes [1], Stefano Dusi [2*], Massimiliano Perduca [1*]

1 Department of Biotechnology, University of Verona, Strada Le Grazie 15, 37134 Verona, Italy.
2 Department of Medicine, Section of General Pathology, University of Verona, Strada Le Grazie 8, 37134, Verona, Italy
* Correspondence: MP, massimiliano.perduca@univr.it, Tel.: +39 0458027984; SD, stefano.dusi@univr.it, Tel.: +39 0458027124

Keywords: dendritic cells, oxyresveratrol, cytokines, PLGA nanoparticles, inflammation

Number of pages: S1-S6
Number of Tables: Table S1-S2
Number of Figures: Figure S1-S3

Table S1. Comparison between DLS, AFM and Nanosight tracking analysis data for empty and oxyresveratrol loaded (PLGA[oxy]) nanoparticles.

Nanoformulation	Z-average (nm)	Peak number (nm)	Afm diameter*(nm)	Nanosight tracking analysis (nm)
Empty	170.2 ± 2.5	148.3 ± 43.0	165.0 ± 34.5	140.8 ± 25.0
PLGA[Oxy]	169.6 ± 3.5	140.5 ± 42.9	171.0 ± 58.7	139.3 ± 26.7

*The statistical analysis was performed over a population major than 100 particles using SPIP™ statistical tool.

Table S2. Comparison between encapsulation efficiency value calculated directly breaking the nanoparticles (EE_{direct}) and the same value indirectly estimated ($EE_{indirect}$). The results are expressed as the mean value of the percentage ±SD of the initial amount of oxyresveratrol added to the reaction for three independent measures on three replica samples.

Nanoformulation	EE_{direct} (%)	$EE_{indirect}$ (%)
PLGA[OXY]	43.75 ± 3.1	45.47 ± 4.55

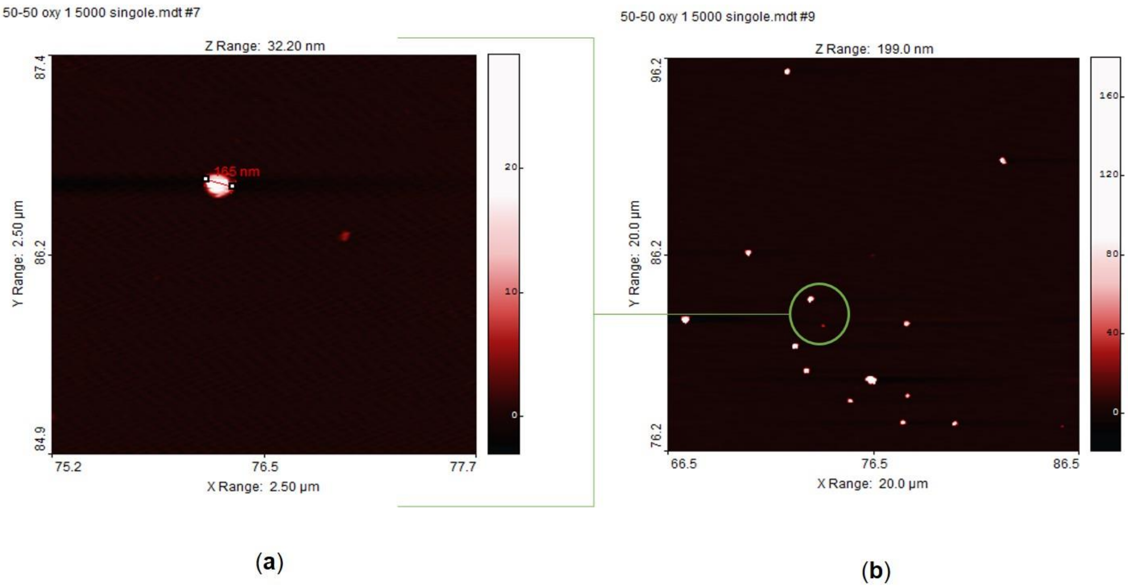

Figure S1. AFM image of a single oxyresveratrol-loaded PLGA nanoparticle with a resolution of 2.5x2.5 um (**a**). Image of several single loaded nanoparticles and an aggregate with a resolution of 20X20 um (**b**). The images were collected in intermittent mode.

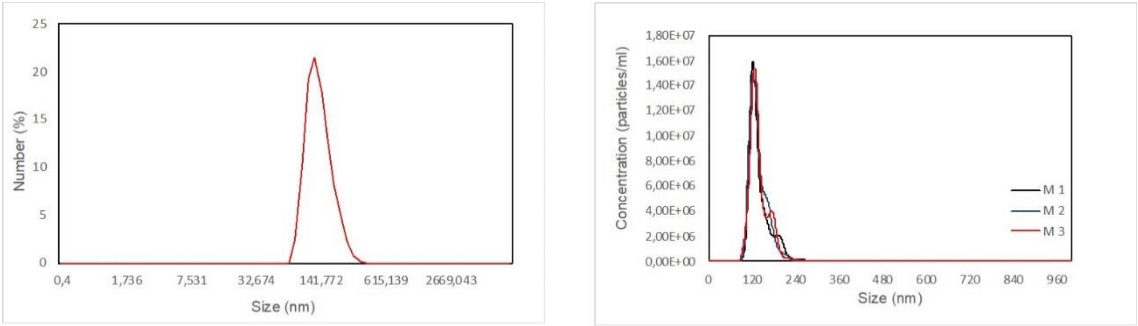

(a) (b)

Figure S2. DLS Number diagram (**a**) and Nanosight tracking analysis diagram (**b**) of oxyresveratrol-loaded PLGA nanoparticles. Measurements where performed in triplicate.

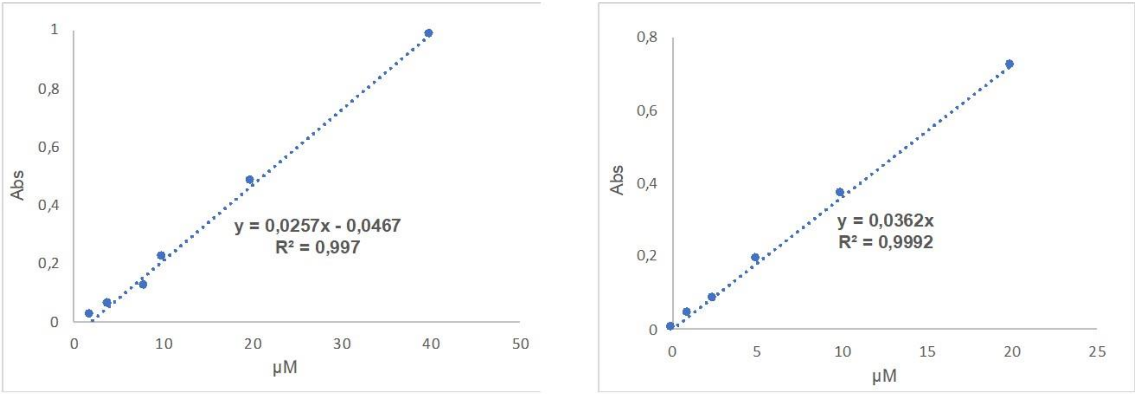

(**a**) (**b**)

Figure S3. Calibration curve for oxyresveratrol in DMSO (**a**). Calibration curve for oxyresveratrol in aqueous solution (**b**).
